# Large Malignant Peripheral Nerve Sheath Tumor Arising From the Sciatic Nerve in a Patient With Neurofibromatosis Type 1

**DOI:** 10.7759/cureus.107751

**Published:** 2026-04-26

**Authors:** Mohammed Benlili, El Abbassi Mohamed, Doha Arreyouchi, Oufkir Ayat Allah

**Affiliations:** 1 Department of Plastic and Reconstructive Surgery, Faculty of Medicine and Pharmacy of Oujda, Mohammed First University, Oujda, MAR

**Keywords:** malignant peripheral nerve sheath tumor, neurofibromatosis type 1, peripheral nerve tumor, sciatic nerve, soft tissue sarcoma

## Abstract

Malignant peripheral nerve sheath tumors (MPNSTs) are rare and aggressive soft tissue sarcomas arising from peripheral nerves or through the malignant transformation of preexisting benign nerve sheath tumors, particularly in patients with neurofibromatosis type 1 (NF1). Sciatic nerve involvement is uncommon and presents major diagnostic and surgical challenges because of the risk of significant functional impairment.

A 21-year-old man with NF1 presented with a one-year history of a rapidly progressive right thigh mass associated with pain and severe motor deficit of the lower limb. Magnetic resonance imaging showed a large heterogeneous intramuscular mass in the posterior compartment of the right thigh, measuring 167 × 100 × 84 mm, with intratumoral necrotic areas and close continuity with the sciatic nerve. Core needle biopsy confirmed MPNST. After active bleeding developed at the biopsy site, urgent surgical resection was performed. Intraoperatively, the tumor was found to involve the sciatic nerve, particularly its common peroneal division, which required sacrifice during excision. A redundant plexiform neurofibroma on the medial aspect of the thigh was also excised separately. Histopathological examination confirmed the Fédération Nationale des Centres de Lutte Contre le Cancer (FNCLCC) grade 3 MPNST with negative surgical margins. The patient subsequently received adjuvant radiotherapy and chemotherapy. At the two-year follow-up, there was no evidence of local recurrence or distant metastasis, and neurological status remained stable with rehabilitation.

This case highlights the diagnostic complexity and surgical difficulty of giant sciatic nerve MPNST in a patient with NF1. Early recognition, complete surgical resection, and multidisciplinary management are essential to optimize oncologic outcomes.

## Introduction

Malignant peripheral nerve sheath tumors (MPNSTs) are rare sarcomas of peripheral nerve sheath origin that may occur de novo or result from the malignant transformation of benign nerve sheath lesions [1,2]. They are clinically important because of their aggressive behavior, high rates of local recurrence, and metastatic potential, all of which contribute to poor long-term outcomes [1,3]. A substantial proportion of cases occur in patients with neurofibromatosis type 1 (NF1), an inherited tumor predisposition syndrome associated with an increased lifetime risk of MPNST and with poorer prognosis compared with sporadic forms [3,4].

MPNST involving the sciatic nerve is rare and presents major diagnostic and therapeutic challenges [5,6]. Because the sciatic nerve is a major motor and sensory structure of the lower limb, tumor involvement at this site raises important concerns regarding both oncologic resection and postoperative functional impairment [5]. In patients with NF1, persistent pain, rapid enlargement of a preexisting lesion, or newly developed neurological deficits should raise suspicion for malignant transformation and prompt urgent evaluation [3,4].

Imaging plays a central role in the assessment of peripheral nerve sheath tumors. Features favoring malignancy include large tumor size, internal heterogeneity, irregular margins, and intratumoral necrosis, whereas benign lesions are typically smaller, better circumscribed, and more homogeneous [7]. We report the case of a giant sciatic nerve MPNST arising in a young adult with NF1, managed by complete surgical excision followed by adjuvant radiotherapy and chemotherapy.

## Case presentation

A 21-year-old man with a known history of NF1, diagnosed on the basis of established clinical criteria, was referred for progressive pain in the right lower limb associated with worsening motor weakness. According to the patient, the lesion had been evolving for approximately one year, with marked acceleration of growth in the months preceding consultation.

**Figure 1:**
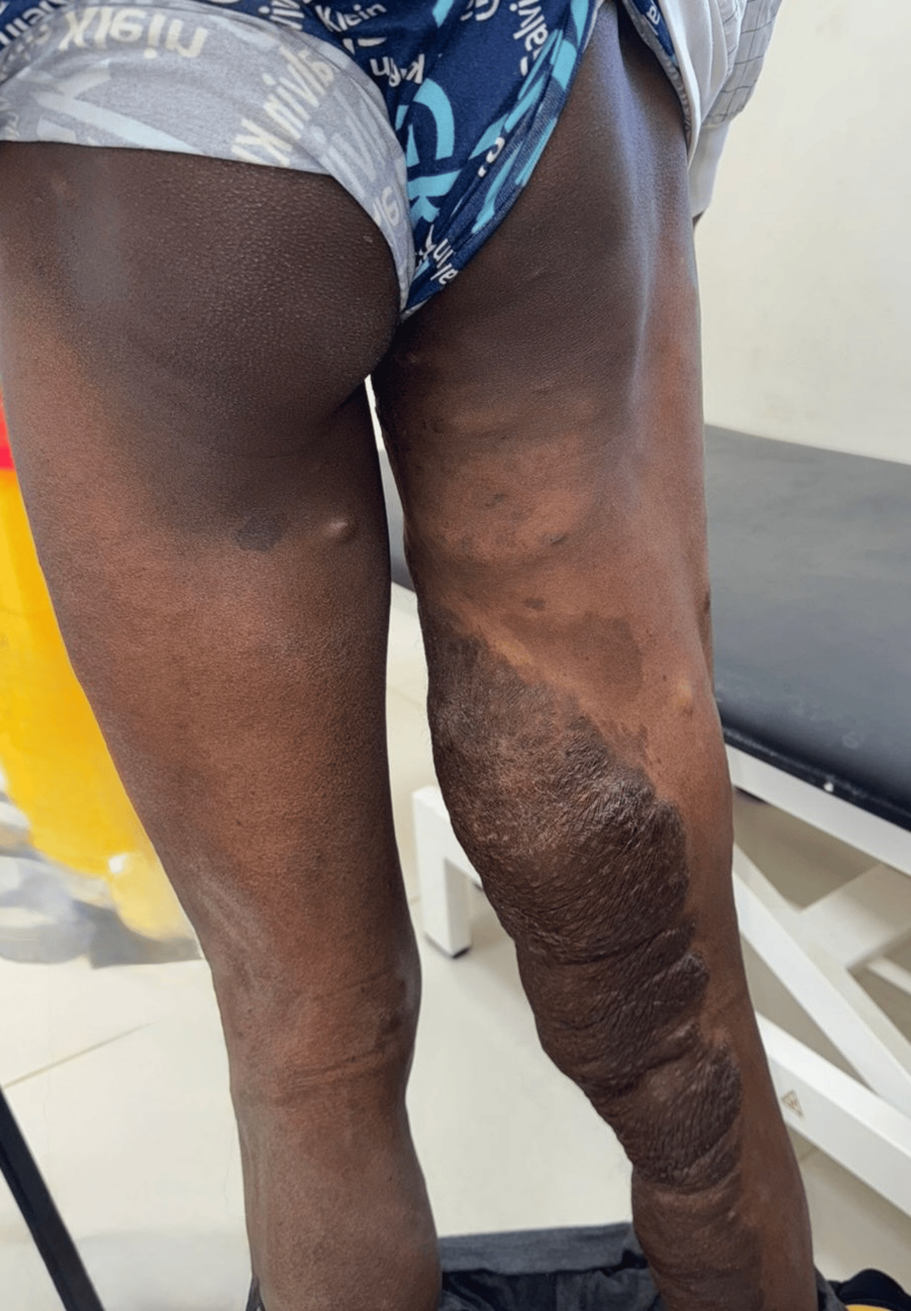
Preoperative clinical appearance of the right thigh showing a large posterior thigh mass with associated plexiform neurofibromatous changes and café-au-lait pigmentation.

On physical examination, a large swelling of the posterior aspect of the right thigh was noted, together with a plexiform neurofibroma along the medial side of the thigh (Figure 1).

Neurological assessment demonstrated severe motor impairment. Both ankle dorsiflexion and plantar flexion were graded at 1/5 using the Medical Research Council (MRC) muscle strength scale. Sensory testing was normal, and tendon reflexes were preserved. No additional neurological abnormalities were identified.

Preoperative laboratory evaluation showed mild anemia, with a hemoglobin level of 11.6 g/dL. White blood cell count and platelet count were within normal limits, serum creatinine was normal, and C-reactive protein was slightly elevated (Table 1).

**Table 1:** Preoperative laboratory findings.

Parameter	Patient value	Unit	Reference range
Hemoglobin	11.6	g/dL	13.0-17.0
White blood cell count	7.2	×10⁹/L	4.0-10.0
Platelet count	322	×10⁹/L	150-400
C-reactive protein	6	mg/L	0-5
Serum creatinine	85	µmol/L	62-106

**Figure 2:**
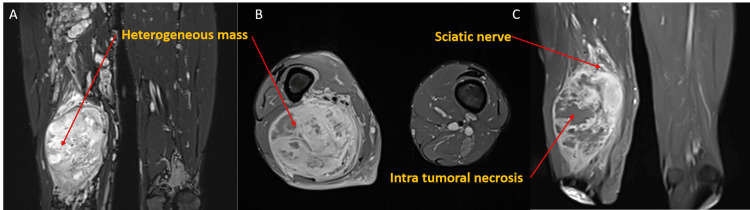
MRI of the thigh showing a malignant peripheral nerve sheath tumor. (A) Coronal fat-suppressed T2-weighted image demonstrating a large heterogeneous soft tissue mass of the thigh with marked craniocaudal extension. (B) Axial post-contrast fat-suppressed T1-weighted image showing heterogeneous enhancement (C) with internal non-enhancing areas suggestive of necrosis. Long-axis post-contrast image further depicting the lesion extent and internal heterogeneity, with the mass appearing in close contact with the sciatic nerve.

Magnetic resonance imaging (MRI) showed a large fusiform soft tissue mass occupying the posteroinferior compartment of the right thigh (Figure 2). The lesion was isointense relative to muscle on T1-weighted sequences and heterogeneously hyperintense on T2-weighted images. Several intralesional necrotic areas were present. The tumor measured 167 × 100 × 84 mm and appeared to be contiguous with the sciatic nerve.

**Figure 3:**
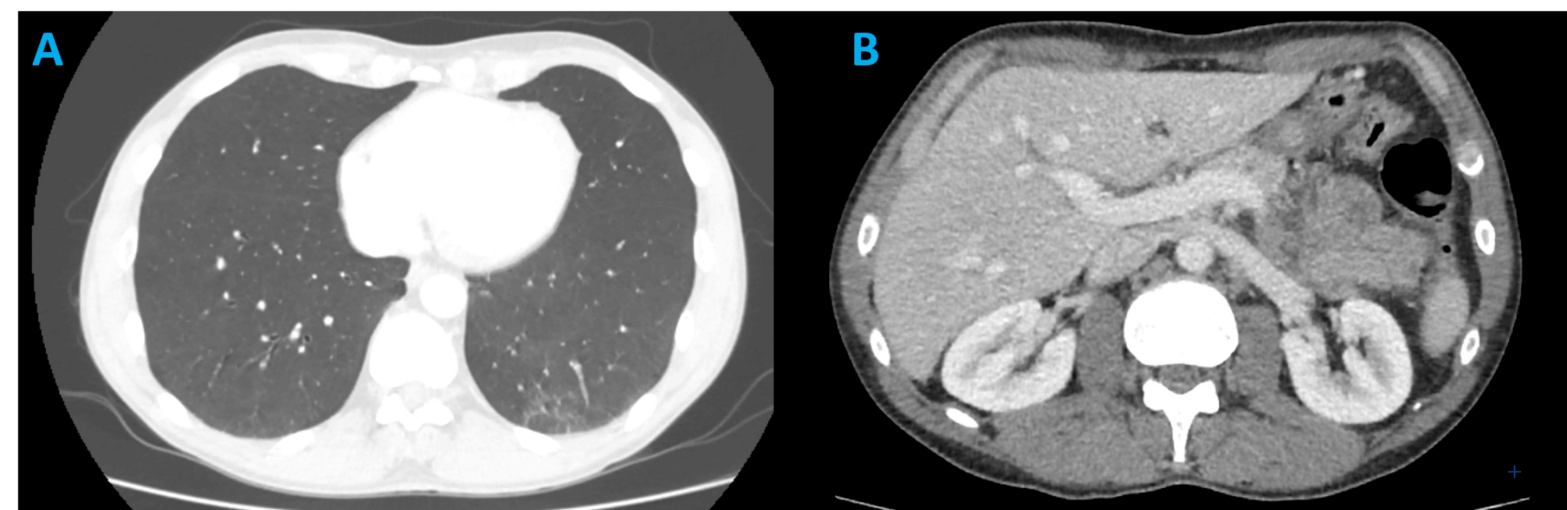
Contrast-enhanced CT scan. (A) Axial chest CT image in lung window showing no evidence of pulmonary nodules or metastatic lesions. (B) Axial abdominal CT image in portal venous phase demonstrating homogeneous liver parenchyma without focal lesions, consistent with the absence of hepatic metastases. CT: computed tomography

A core needle biopsy was performed under ultrasound guidance. Histological evaluation was consistent with MPNST. A thoraco-abdomino-pelvic computed tomography (CT) workup did not identify any distant metastatic lesions (Figure 3).

Shortly after the biopsy, the patient developed active bleeding from the puncture site, which precluded the completion of the planned electroneuromyography (ENMG) and prompted urgent surgical management. The case was subsequently discussed at a multidisciplinary sarcoma board meeting, and surgical excision was indicated.

**Figure 4:**
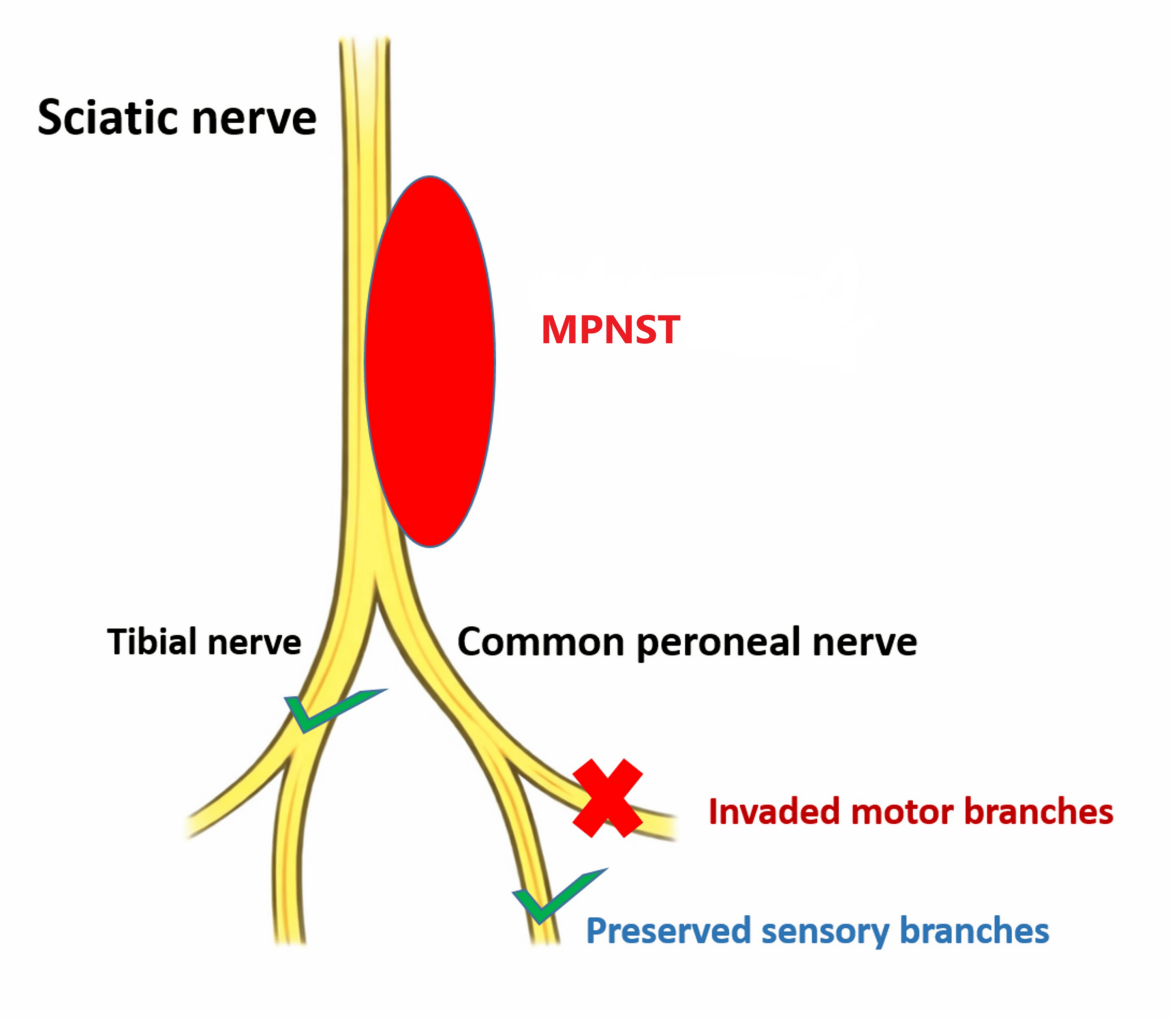
Schematic representation of the sciatic nerve and its terminal branches. The tumor arose from the motor branches of the common peroneal nerve. Tumor resection required the excision of the involved motor branches, while the distal uninvolved branches were preserved. MPNST: malignant peripheral nerve sheath tumor This illustration was created using Microsoft PowerPoint (Microsoft Corp., Redmond, WA, USA).

At surgery, the tumor occupied most of the posterior compartment of the thigh and displaced the surrounding musculature. The anterior surface of the mass was readily dissected and was found to be clearly separable from the femur. Posteriorly, dissection between the lesion and the semimembranosus muscle revealed a markedly hemorrhagic cleavage plane. The inferior pole of the tumor was then exposed and was seen to extend distally into the common peroneal division of the sciatic nerve. The surgical anatomy and distal nerve preservation are illustrated in Figure 4.

Complete separation of the tumor from the sciatic nerve was not feasible because of direct tumoral infiltration. Intraoperative bleeding was profuse and originated from the abnormal vascularization of the involved nerve, requiring the application of a tourniquet. A portion of the common peroneal division was ligated and resected, revealing pathologically dilated vessels and abnormal nerve fascicles. Because of direct neural invasion, sacrifice of this nerve segment was necessary to achieve complete oncologic excision. Nerve grafting had been considered preoperatively in anticipation of possible nerve sacrifice. However, intraoperative findings, particularly the markedly abnormal and dilated appearance of the nerve fascicles, made nerve reconstruction unsuitable. In addition, the patient's hemodynamic instability did not support the prolongation of the procedure for grafting.

**Figure 5:**
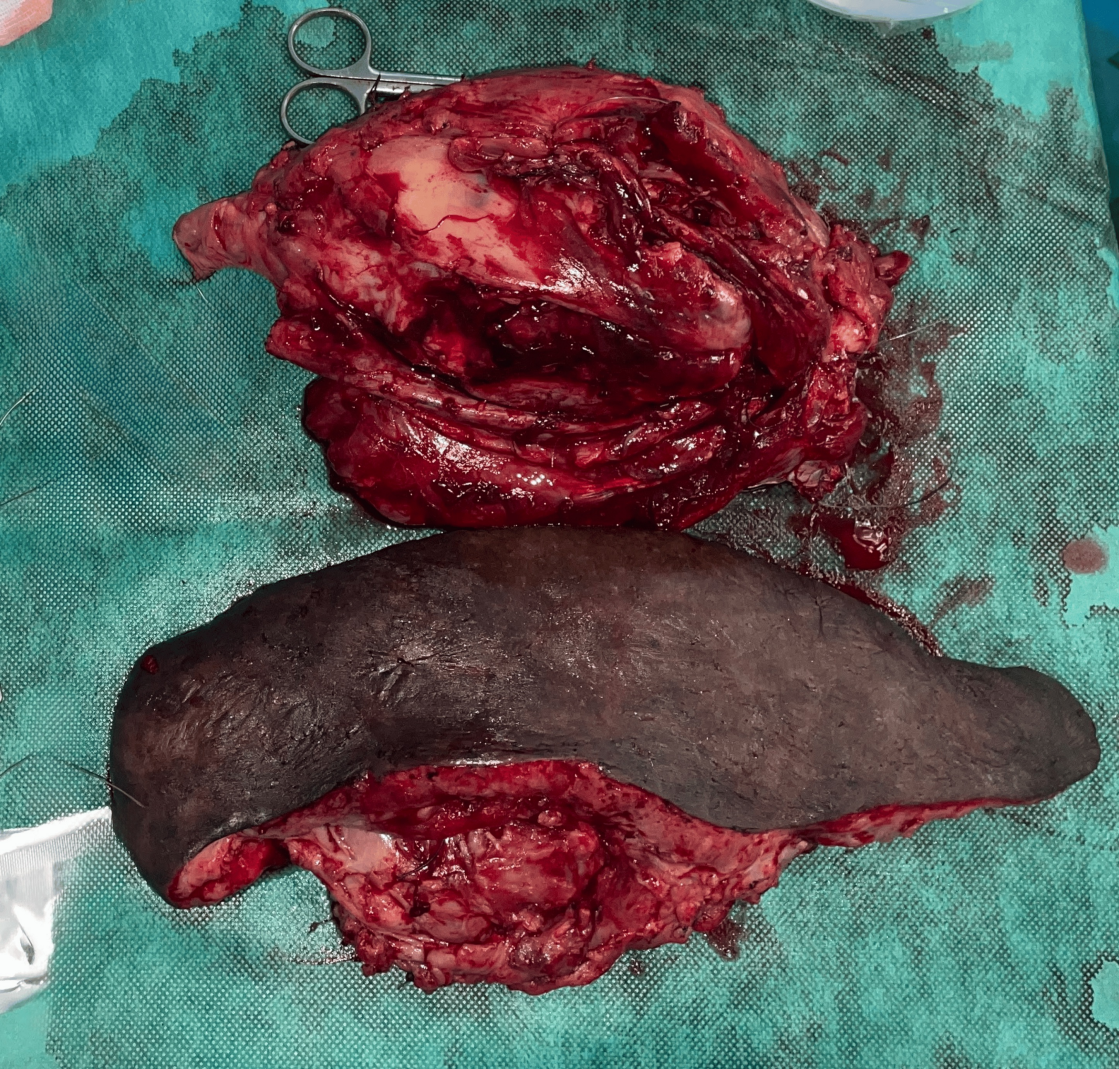
Operative specimen after tumor resection. The surgical specimen shows the resected thigh soft tissue tumor (upper panel) and the neurofibromatous skin excised separately (lower panel).

The anticipated postoperative deficit was permanent foot drop, mainly consisting of impaired ankle dorsiflexion, loss of toe extension, and weakness of foot eversion. As postoperative sensory examination remained normal, the neurological deficit was considered to predominantly affect the motor component of the common peroneal division. Rehabilitation included ankle-foot orthosis fitting, supervised gait training, preservation of ankle range of motion, strengthening of preserved muscle groups, and preventive measures against contracture and falls. A redundant plexiform neurofibroma located on the medial aspect of the thigh was also excised during the same procedure (Figure 5). This excess plexiform tissue provided sufficient soft tissue coverage and allowed tension-free, hermetic wound closure without the need for additional reconstructive procedures.

**Figure 6:**
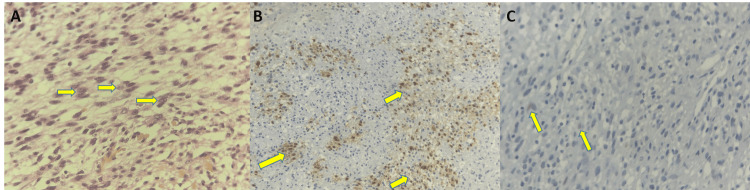
Histopathological and immunohistochemical findings. (A) H&E, ×40, showing atypical spindle cells with mitotic activity. (B) S100: focal immunoreactivity in tumor cells. (C) SOX10: focal tumor cell positivity. H&E: hematoxylin and eosin

Macroscopic excision was complete. Final pathological analysis confirmed a grade 3 MPNST according to the Fédération Nationale des Centres de Lutte Contre le Cancer (FNCLCC) grading system. The tumor showed 15 mitoses per 10 high-power fields, with necrosis involving less than 50% of the lesion. An immunohistochemical study demonstrated the focal expression of S100 protein and SOX10, supporting neural differentiation. The Ki-67 proliferative index was increased. Surgical margins were free of tumor (Figure 6).

After surgery, no new neurological deficit beyond the preoperative condition was observed. The patient was referred to a rehabilitation program including physical therapy and management of foot drop.

**Figure 7:**
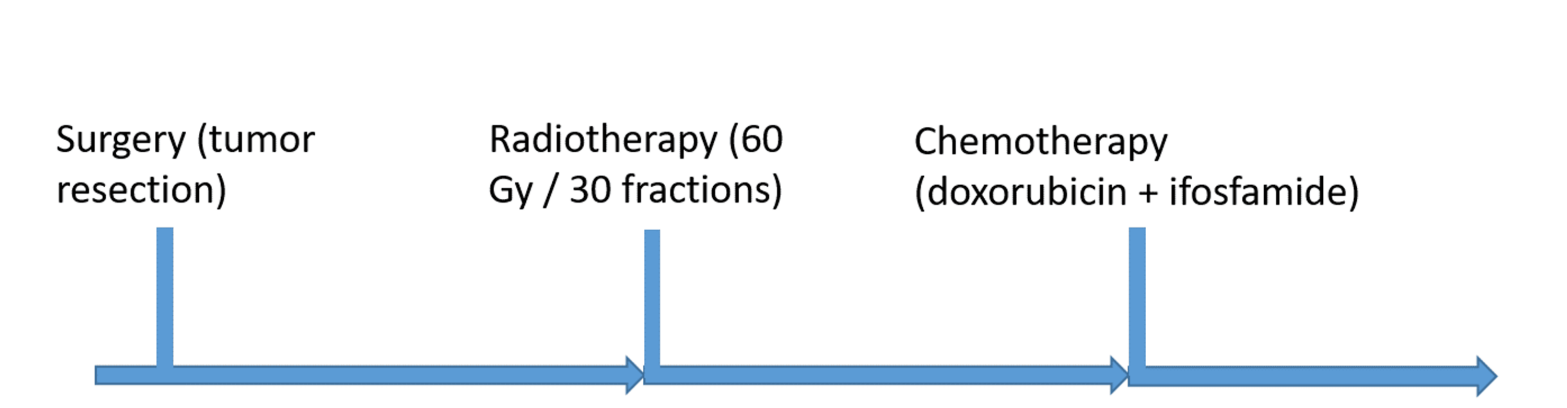
Treatment timeline. The patient underwent surgical resection, followed by adjuvant radiotherapy delivered at a total dose of 60 Gy in 30 fractions, and then chemotherapy with doxorubicin and ifosfamide.

Adjuvant treatment consisted of external beam radiotherapy delivered to a total dose of 60 Gy in 30 fractions, followed by chemotherapy with doxorubicin and ifosfamide. The sequence of multimodal treatment is summarized in Figure 7.

At two years of follow-up, clinical examination and imaging showed no evidence of local recurrence or distant metastasis. Neurological function remained stable, without further deterioration.

## Discussion

MPNST is an uncommon but highly aggressive sarcoma of peripheral nerve sheath origin and is associated with a generally poor prognosis [1,3]. Outcome is influenced by several factors, particularly tumor size, histological grade, surgical margin status, and the presence of metastatic disease at diagnosis [1,3]. In the setting of NF1, malignant transformation frequently develops from preexisting plexiform neurofibromas and often affects younger patients than sporadic MPNST, which is consistent with the age and background of our patient [4,6].

The present case is notable because of the combination of NF1, the exceptional size of the tumor, and the involvement of the sciatic nerve, an uncommon location associated with major technical and functional challenges [5,6]. The clinical presentation strongly suggested malignant transformation, as the patient developed progressive pain, rapid enlargement of the lesion, and marked motor deficit, all of which are considered warning signs in NF1-associated peripheral nerve sheath tumors [3,4].

Radiologically, the lesion demonstrated several features suggestive of malignancy, including large size, heterogeneous signal intensity, and intratumoral necrosis [7]. These findings are in agreement with published MRI descriptions distinguishing MPNST from benign peripheral nerve sheath tumors, which are usually smaller, better defined, and more homogeneous. The main comparative MRI features are summarized in Table 2 [7]. In our patient, the close continuity between the lesion and the sciatic nerve further reinforced the suspicion of MPNST before histopathological confirmation.

**Table 2:** Comparative MRI features of benign peripheral nerve sheath tumor and MPNST. MPNST: malignant peripheral nerve sheath tumor; MRI: magnetic resonance imaging

MRI feature	Benign peripheral nerve sheath tumor	MPNST
Size	Usually small to moderate	Often large
Margins	Well defined	Irregular or ill-defined
Signal intensity	More homogeneous	More heterogeneous
T2 appearance	Usually homogeneous hyperintensity	Heterogeneous hyperintensity
Intratumoral necrosis	Uncommon	Frequent
Adjacent tissues	Usually displaced	May be infiltrated

Histopathological examination remains essential for definitive diagnosis. In the present case, the tumor consisted of a high-grade spindle cell malignant proliferation with increased mitotic activity and necrosis. Immunohistochemistry demonstrated focal positivity for S100 protein and SOX10. This finding is important because MPNST often shows only focal or patchy expression of Schwannian markers, in contrast to benign Schwannian tumors, in which staining may be more diffuse. Focal S100 positivity therefore supports neural differentiation but must be interpreted together with morphology and clinical context. SOX10 is particularly useful in this setting, as it supports Schwannian differentiation and may help distinguish MPNST from other spindle cell sarcomas, especially synovial sarcoma [8]. Kang et al. showed that SOX10 has diagnostic utility in differentiating MPNST from synovial sarcoma, which is an important histologic mimic [8]. In our case, the combination of spindle cell morphology, high-grade features, nerve continuity, focal S100 positivity, and SOX10 expression supported the diagnosis of MPNST over alternative spindle cell neoplasms.

Surgical excision with negative margins remains the cornerstone of treatment for localized MPNST and the main determinant of local disease control [1,3]. However, complete resection may be particularly challenging in sciatic nerve tumors because adequate oncologic clearance can require sacrifice of functional neural tissue [5,6]. In the present case, direct tumoral infiltration required the resection of the motor branches of the common peroneal division, while the uninvolved distal branches were preserved. The expected consequence was permanent foot drop with loss of ankle dorsiflexion and toe extension, which justified early postoperative rehabilitation and ankle-foot orthosis fitting as part of the overall treatment strategy.

Adjuvant radiotherapy is frequently used in high-grade or large tumors to improve local control, whereas chemotherapy may be considered in selected high-risk cases, especially in young patients with large, deep, high-grade lesions [3,9]. Our patient received postoperative radiotherapy and doxorubicin-ifosfamide chemotherapy as part of a multidisciplinary treatment strategy.

This report has limitations. First, it describes a single case, which limits the generalizability of the findings. Second, although no recurrence or metastasis was observed during the first two postoperative years, this follow-up remains relatively short for a tumor known to carry a risk of late local recurrence and delayed metastatic progression [1,3]. Continued long-term surveillance is therefore mandatory.

## Conclusions

In patients with NF1, the occurrence of persistent pain, rapid increase in lesion size, or newly developed neurological deficit should raise suspicion for MPNST. The present case illustrates the diagnostic and therapeutic challenges posed by a giant sciatic nerve MPNST. Achieving negative margins may require major functional sacrifice, making multidisciplinary planning essential. Early diagnosis, complete oncologic resection, adjuvant treatment when indicated, and prolonged follow-up are key elements in the management of these tumors.
